# Stage-Dependent Effects of Rapamycin for Alzheimer’s Disease: Insights from Prevention and Treatment Trials

**DOI:** 10.1093/geroni/igaf122.405

**Published:** 2025-12-31

**Authors:** Mitzi Gonzales, Ai-Lin Ling

**Affiliations:** Cedars-Sinai Medical Center, Los Angeles, California, United States

## Abstract

Rapamycin is the best-established longevity-promoting drug in animal models and has been shown to attenuate Alzheimer’s disease pathology in preclinical trials. Here we compare the findings of a prevention versus therapeutic clinical trial of rapamycin for Alzheimer’s disease and discuss implications for applying the geroscience hypothesis to Alzheimer’s disease. Two open-label pilot studies evaluated the safety and preliminary efficacy of short-term (4-12 week) low dose (1 mg/day) rapamycin treatment. The prevention trial enrolled cognitively unimpaired middle-aged APOE4 carriers and non-carriers, whereas the therapeutic trial enrolled older adults with mild cognitive impairment or dementia. Both studies examined changes in plasma inflammatory markers. The prevention study also examined cerebral blood flow, and the therapeutic study assessed Alzheimer’s disease biomarkers in cerebrospinal fluid (CSF). Baseline to post-treatment changes were evaluated using paired samples t-tests or Wilcoxon signed rank tests. The prevention study enrolled 9 APOE4 carriers (mean age 53 ± 8 years, 44% female) and 14 APOE-4 non-carriers (mean age 55 ± 6 years, 50% female). APOE4 carriers demonstrated reduced plasma cytokines and increased cerebral blood flow at post-treatment (all p < 0.05) that were not observed in non-carriers. In contrast, participants in the therapeutic trial displayed increased plasma cytokines and CSF phosphorylated tau 181, neurofilament light, and glial fibrillary acidic protein levels (FDR-corrected p-value<0.05). Overall, the results of the two trials suggest that rapamycin may have markedly divergent effects on inflammatory and Alzheimer’s disease biomarkers when administered for prevention versus treatment, highlighting the importance of tailoring geroscience-informed interventions for the appropriate disease stage.

